# Validation and Psychometric Analysis of the German Translation of the Appraisal of Self-Care Agency Scale-Revised

**DOI:** 10.3390/healthcare10091785

**Published:** 2022-09-16

**Authors:** Aline Schönenberg, Ulrike Teschner, Tino Prell, Hannah M. Mühlhammer

**Affiliations:** 1 Department of Geriatrics, Halle University Hospital, 06120 Halle, Germany; 2 Department of Neurology, Jena University Hospital, 07747 Jena, Germany

**Keywords:** self care, self management, chronic illness, health care, self-efficacy, older adults

## Abstract

Self-care and self-management are essential for well-being, especially in advancing age or chronic illness. To assess these complex behaviors, validated questionnaires are needed. The Appraisal of Self-Care Agency Scale-Revised (ASAS-R) is a self-report questionnaire to evaluate the actions people take to manage their health. This manuscript reports the psychometric properties of the German ASAS-R translation. After standardized translation, convergent validity was assessed with the Patient Activation Measure (PAM) controlling for sociodemographic and health factors. Internal consistency, descriptive statistics, and principal component analysis (PCA) are reported. We analyzed data of 215 community-dwelling German adults aged 51.6 ± 14.7 years with at least one chronic illness. Similar to the original ASAS-R, PCA revealed three factors, although item allocation differed. The ASAS-R showed good internal consistency overall and for each factor, although ceiling effects were present for some items. Convergent validity was good, and the ASAS-R was as a predictor for the PAM irrespective of other variables. As self-care is highly complex, we conclude that factor structure should be assessed for each dataset. Overall, the German ASAS-R is a valid instrument to measure self-care and self-management of chronic diseases that may enhance research on this fundamental health behavior in German-speaking countries.

## 1. Introduction

Patients are often expected to deal with complex conditions such as multiple medications, fluctuating health and symptoms, or age-related changes. Especially in advancing age or with chronic illness, where patients are confronted with health issues on a daily basis, the concept of self-care and self-management is of utmost importance [1,2]. As Udlis [3] stated, “people cannot *not* self-manage” as every choice made, whether regarding physical activity, diet, medication, social interaction, or further domains, is a type of self-management. Self-management is a complex construct that describes the ability to appropriately handle health-related changes (including symptom monitoring, detection of changes, and appropriate responding), manage medications, and perform appropriate physical activities and diets. However, self-management also includes psycho-social aspects, such as a general belief in the ability to successfully perform changes (self-efficacy, [4,5,6]), adaptation of social roles, maintenance of social interaction, problem solving, and emotion regulation [1,3,4,7,8,9]. A key requirement for self-management is active patient participation [3,7], meaning that self-management refers to daily measures and actions performed by the patients themselves [10].

Self-care is a closely related concept and has been defined as a crucial cornerstone for health by the World Health Organization (WHO) [11,12]. Self-care agency, meaning the drive and ability to perform self-care actions, is closely linked to increased health-improving behavior and thus to self-management of chronic illness [13,14]. As defined by the WHO, “self-care interventions promote the active participation of individuals in their healthcare”, defining self-care as a building block of self-management [3]. Likewise, in their 2022 revision of their guidelines for self-care interventions, the WHO suggests self-management as a more active part of self-care [12], see also Narasimhan et al. (2019) [15] and Matarese et al. (2018), who describe self-management as a related or even surrogate term for self-management [16].

Due to this close relation, many questionnaires designed to measure self-management are often based on self-care agency [4]. Riegel et al. (2009) even introduced the term “self-care management” as a combination of both constructs [14], describing is as “decision making in response to signs and symptoms”.

Overall, self-care and subsequently self-management are key components of successful healthcare, especially in the face of demographic change and the increase of chronic [11,17,18]. Poor health outcomes, increased health care costs, and adverse health events are associated with an ineffective utilization of self-management strategies at home [1,2]. In contrast, effective self-care and self-management are associated with better health outcomes, reduced healthcare utilization (especially re-hospitalization), and overall better quality of life (QoL) [1,19,20].

Therefore, measuring self-management is a crucial step in understanding the factors pertaining to this aspect of healthcare. Thus far, several questionnaires have been developed to measure self-management and self-care [4,21,22,23], but none of them cover all the abovementioned aspects of this complex health behavior. Thus, it is recommended to use multiple tools to measure self-care and self-management [4,24].

The Appraisal of Self-Care Agency Scale-Revised (ASAS-R) is built around Orem’s Theory of Self-Care [25], which highlights the need to maintain some degree of independence and self-care agency even when faced with serious health issues. For this purpose, it is essential to consider a patients’ personal situation and to identify the individual needs and acts of self-care that can still be performed [25,26]. The questionnaire was initially developed by Evers et al. [27] and revised by Sousa et al. [28]. It has been translated into different languages and tested in various settings, showing overall good psychometric properties [29,30,31,32]. By its name, it is not strictly defined as a self-management tool; however, its items correspond to the content of self-management, and self-care is a key component of self-management. Therefore, the ASAS-R is suitable as a tool to measure self-management [28,32]. Of special interest, some items in the ASAS-R are worded in a way that refers to behavior instead of hypothetical beliefs, e.g., “I *make* needed adjustments”, “in the past, I *have*…”, or “I *regularly* evaluate…”. For example, the six-item Self-Efficacy for Managing Chronic Disease Scale (SES6G) [33,34] explicitly asks about *confidence* in the ability to perform self-management, but not about the actual performance itself. Of note, asking about *beliefs* may not be enough when predictors for actual self-management must be identified or when the interest lies in the improvement of self-management that must be measured in its actual execution, for example, in an intervention trial. To date, no German version of the ASAS-R is available; however, due to its promising psychometric properties and its focus on *actions* rather than solely *beliefs*, the ASAS-R is a useful questionnaire to add to the list of self-management measures. Therefore, we aimed to translate the questionnaire into German and validate the translation to make it available for use in German-speaking countries.

## 2. Materials and Methods

### 2.1. Translation

The English ASAS-R questionnaire was forward-translated into German by a German native speaker, who is both highly proficient in English and knowledgeable about the field of scientific research and healthcare. The German version was then translated back into English by a second person, also a native German speaker with English proficiency on native speaker level and a high level of knowledge in healthcare and social sciences. In addition, another independent researcher who is not part of the study team but still knowledgeable about the related concepts and highly proficient in English further translated the original questionnaire into German. Afterward, the translated German versions were compared, and the re-translated English version was compared with the original ASAS-R. Slight variations in the translations were discussed between the researchers with regards to their potential influence on the content of each item until consent was reached [35]. The translated version of the questionnaire is given in the Supplementary Materials (Supplementary Table S1).

### 2.2. Data Collection and Participants

Data were collected between April and July 2022. Inclusion criteria were comprised of age ≥ 18, the presence of at least one chronological disorder that had been diagnosed for at least 1 year, and the absence of severe depression or dementia.

Overall sample sizes for questionnaire translations have varied greatly, with many studies recruiting between 200 and 300 participants [33,36,37,38,39]. As recommended by Sousa et al. (2010), a sample size of around 10 subjects per item of the questionnaire is required for psychometric analyses [35,40,41], resulting in a minimum of 150 participants for translation of the ASAS-R. Considering potential dropouts due to missing data, our aim was to collect at least 200 datasets.

Participants were contacted via flyers in the hospital and via social media visible to all patients in the hospital. A certificate of non-objection from the local ethics committee of Jena University Hospital (approval number 2022-2515) was obtained. Due to the restrictions of the COVID-19 pandemic, data collection was performed online using the tool SoSciSurvey [42]. This way, patients were able to fill out the questionnaire on their mobile phones while waiting for their appointment or could take a flyer home with them to fill it out on their computers. Thus, all information collected was based on self-report. First, information about the aim of the study and data protection was presented, and participants gave explicit consent to participate. Afterward, sociodemographic and health-related information were collected:

- *Age* in years, *marital status* (married, single, widowed/divorced), *education* as corresponding to the German education system (low: no school to 8 years, medium: 10 years, high: a-levels (at least 12 years) or higher educational degree)
- *Number* and type of *diagnoses*, selected from a list of 15 choices as specified in the Survey of Health, Ageing and Retirement in Europe (SHARE) dataset (http://www.share-project.org/home0.html, accessed on 29 July 2022) and the option to add further diagnoses, and year of main diagnosis to calculate the variable *disease duration* (2022—answer given in Year of Diagnosis)
- *Number of medications* taken daily
- Restrictions in daily activities (*ADLs*) based on the item used in the DEAS dataset, a large nationwide assessment of elderly patients in Germany: “In the last 6 months or longer, have you been restricted in your daily activities for health-related reasons?” (1 = yes, strongly, 2 = yes, a bit, 3 = no) [43]
- Item 1 of the SF-36 to assess *current health* status (Would you say your current health is… (1) excellent, (2) very good, (3) good, (4) fair, (5) poor) [44,45]

For convergent validity, the German version of the Patient Activation Measure (PAM, short form) was used, which is a one-factorial questionnaire comprised of 13 items on a 4-point Likert scale ranging from 1 = completely disagree to 4 = completely agree [46,47,48]. The PAM is a widely used measure of patient initiative and self-care [46,48,49]; however, it mainly inquires after *hypothetical* behavior and not after measures that have already been taken. A sum score is calculated for the PAM by adding up the values of the individual items.

The translated version of the ASAS-R was the main measurement of interest. It comprises 15 items posed on a 5-point Likert scale ranging from 1 = totally disagree to 5 = totally agree. Items 4, 11, 14, and 15 of the ASAS were reverse-coded and thus re-scaled to match the scale of the other items. A three-factor model has been proposed for the revised version, measuring the sub-components *having* power for self-care*, developing* power for self-care*,* and *lacking* power for self-care [28]. To calculate an overall sum score as well as the scores for the sub-scales, the corresponding item answers are added up, with higher scores indicating higher self-care agency.

Since the PAM and the ASAS-R contain similar items, their order within the questionnaire was randomized between participants. This was done to ensure that answers from one questionnaire did not needlessly influence answers from the other, e.g., due to frustration or confusion regarding the similar questions.

Due to the online format, participants were instructed to conduct the questionnaire in a quiet setting without distractions. Although it was not possible to objectively measure whether participants adhered to this, they were asked at the end to indicate whether they completed the questionnaire (1) in a quiet setting without distractions, (2) in a quiet setting with minimal distractions (1–2 times), or (3) with many distractions. In between the PAM and the ASAS, we furthermore included a control item instructing the participants to select a particular option on the presented scale to ensure that participants read the items and instructions carefully instead of blindly selecting random answers. These two items were not included in the analysis. Instead, they simply served as control items, and participants who failed to select the correct response or who admitted to being distracted many times were excluded from the analysis. In addition, participants who selected “none” when asked to indicate their chronic diagnoses and participants who failed to respond to one or several items of ASAS and PAM were also excluded from the analysis.

### 2.3. Statistical Analysis

All statistical analyses were performed in R Version 4.1.1 [50]. As a first step, descriptive statistics (mean with standard deviation (SD), as well as median and interquartile range (IQR) for metric and count with percentages for categorical variables) were calculated to present the characteristics of the included participants.

Next, the German ASAS-R was assessed in its internal consistency and scale properties via inter-item correlations using Spearman Rank correlations and Cronbach’s Alpha (α), floor and ceiling effects, as well as skew and kurtosis [51]. Floor and ceiling effects were considered present if at least 15% of the respondents reached the lowest (floor) or highest (ceiling) possible item score. Internal consistency was measured with the *psych* package using Cronbach’s Alpha (α) and considered high for values greater than 0.80, with 0.70 being the minimum correlation [51,52,53,54]. Inter-item and item-to-rest correlations were assessed with Spearman correlation (ρ), with correlations of ≥0.10 considered low, ≥0.3 moderate, and ≥0.5 as strong correlations [55]. As recommended, inter-item correlations should be between 0.3 and 0.7 for subscales [56]. As the original publication of the ASAS-R proposes a three-factor structure, confirmatory factor analysis (CFA) was performed using the R-package *Lavaan* [57] to assess whether the proposed structure fits our data. Afterward, Principal Component Analysis (PCA) was performed using the *stats* package [45] to find the best-fitting factor structure for the present data. As the items stem from a single questionnaire measuring an overall latent construct, PCA was performed using oblique oblimin rotation [58,59]. All factors had an Eigenvalue > 1. Requirements for performing factor analysis were assessed using *EFAtools* [60].

To assess the validity of the German ASAS-R, convergent validity was measured with the PAM based on Spearman correlations (*r*) between the sum scores of the two questionnaires. As recommended by Prinsen et al. (2018) [52], correlations between instruments measuring the same constructs should be greater than or equal to 0.5, while correlations for instruments measuring similar but not identical constructs should lie between 0.3 and 0.5. Therefore, a correlation between 0.3 and 0.5 was expected between the PAM and the German ASAS-R.

Finally, linear regression models were performed to assess the association between the PAM and the German ASAS-R and to control for the potential influence of sociodemographic or health-related factors. Therefore, in a first model, the influence of the ASAS-R on the PAM alone was assessed. This model was compared to a second model, including the ASAS-R as well as sociodemographic and health-related information, to predict the PAM. Normal distribution was assessed using the Shapiro–Wilk test and requirements for the linear regression, such as linearity, absence of multicollinearity and autocorrelation, homogeneity of variance, and normality of residuals, were assessed with the *performance* package in R [61]. Based on Cook’s distance, no outliers were detected. As heteroscedasticity was present for Model 1 (Breusch-Pagan Test *p* = 0.037), HC3-type robust coefficients were calculated [62].

*p*-values below 0.05 denote statistical significance, and 95% confidence intervals (CIs) are given wherever appropriate. All visualizations were computed using *ggplot2* [63].

## 3. Results

### 3.1. Participants

In total, *n* = 278 participants filled out the online questionnaire. Of those, 35 participants were excluded from the analysis because they did not complete the questionnaires of interest (*ASAS* and *PAM*). Of the remaining 243 participants, 28 participants were further excluded because they did not respond correctly to the control items as described in the Methods section. In total, *n* = 215 datasets were retained.

Overall, the remaining participants (70% female) had a mean *age* of 51.633 years (SD = 14.686), took an average of 3.219 *medications per day* (SD = 2.802), and reported an average of 1.986 different chronic *diagnoses* (SD = 1.190), with a mean *disease duration* of 13.7784 years (SD = 11.071). The most common diagnoses were hypertension (*n* = 75), chronic obstructive pulmonary disease (*n* = 38), and diabetes (*n* = 34), see Supplementary Table S2 for details. Most participants rated their current *health* as good or not good and reported to be moderately restricted in their *ADLs*. Detailed sociodemographic data are given in Table 1.

### 3.2. Properties of the ASAS-R

All participants included in the analysis completed the ASAS questionnaire without missing items (*n* = 215). Properties of the ASAS are given in Table 2. The Shapiro–Wilk test revealed a moderate non-normal distribution (W = 0.983, *p* = 0.011) with a slight skewness to the left (skew = −0.42, kurtosis = 0.85, SE = 0.57). The mean inter-item correlation was 0.28 for the entire ASAS scale. The ASAS scores did not differ depending on gender (*p* = 0.464, r = 0.05) or education level (*p* = 0.169, eta^2^ = 0.007).

Internal consistency measured by Cronbach’s Alpha for all ASAS-R items was α = 0.82 (95% CI [0.79; 0.86]), indicating a high correlation. Likewise, the corrected item-to-total correlation was above 0.38 for all items (see Table 2) and even above 0.5 for 8 of the 15 items, indicating medium to high correlations. The correlation matrix for all ASAS items is given in Supplementary Figure S1.

However, based on the original publication, a three-factor structure should underlie the questionnaire, so these correlations must be interpreted with caution. In the next step, we therefore aimed to assess if this factor structure could be replicated with our data.

Using CFA, the original ASAS-R structure and item assignment were tested in the present dataset. However, although the model was statistically significant (𝜒^2^ = 392.62, *p* < 0.001), the original model fit poorly to our data (CFI = 0.798, TLI = 0.756, AIC = 8420.25, RMSEA = 0.11, RMSEA *p* < 0.001, see Supplementary Table S3). Therefore, PCA was performed to find the underlying factor structure for the present data. Kaiser-Meyer-Olkin (KMO) criterion (0.826) and Bartlett test (𝜒^2^(105) = 1133.43, *p* < 0.001) suggested that the data were suitable for factor analysis. Based on an Eigenvalue > 1, a three-factor solution, as proposed in the original publication, was suggested. However, the item attribution to the factors differed from the original proposition (see Table 3 and Supplementary Figures S2 and S3).

The resulting model was significant (𝜒^2^ = 191.65, *p* < 0.001), explaining 54.4% of the total variance (see Supplementary Table S4). Using CFA to confirm model fit, the 𝜒^2^ test revealed a significant improvement over the baseline model (𝜒^2^ = 155.563, *p* < 0.001). The model fit the data well, with CFI = 0.92, TLI = 0.903, AIC = 7721.316, and RMSEA = 0.072 ([CI 0.056, 0.087], *p* = 0.014).

The 𝜒^2^-test revealed a better fit for the current than the original model (*p* < 0.001). The three factors had sufficient internal consistency (see Table 2), and the mean inter-item correlation was between 0.3 and 0.44 for all subscales (see Supplementary Table S5).

### 3.3. Comparison of ASAS and PAM

**Figure 1:**
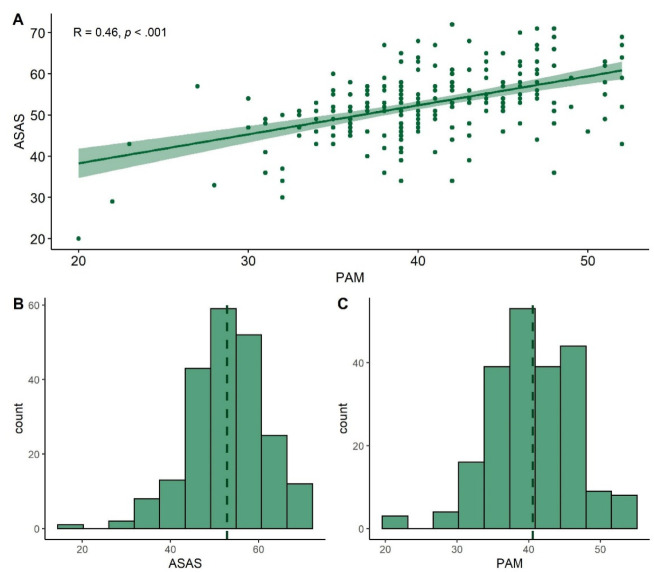
Relationship (**A**) and distribution of ASAS (**B**) and PAM (**C**).

To assess the convergent validity of the ASAS, the correlation between the sum scores of the ASAS and PAM was calculated. The correlation between the sum of ASAS and PAM was ρ = 0.46 (CI [0.35, 0.56], *p* < 0.001), see Figure 1A. Both questionnaires were similarly distributed (Figure 1B,C).

To confirm the relation between ASAS-R and PAM, linear regression models were conducted.

The simple first model (Model 1) contained only the ASAS-R as a single predictor for the PAM sum score. Model 1 was statistically significant (adjusted R^2^ = 0.261, F(1, 204) = 73.45, *p* < 0.0001, AIC = 1264.60). Thus, the ASAS-R alone explained 26% of the variance of the PAM. The ASAS-R was significant as a predictor of the PAM (ß = 0.37, CI [0.29, 0.46], *p* < 0.0001).

Model 2 (Table 4) then contained sociodemographic and health-related information to assess whether the relation between ASAS-R and PAM was independent of further variables. Model 2 was also significant (adjusted R^2^ = 0.438, F(14 191) = 12.41, *p* < 0.0001, AIC = 1220.65) and model comparison via AIC and ANOVA indicated that Model 2 was a better fit for the data (*p* < 0.0001) than Model 1. Model 2 again revealed the ASAS-R as a significant predictor of the PAM.

Likewise, *disease duration*, high *education* level, and the different levels of *health* were identified as significant predictors of the PAM. Therefore, a third regression (Model 3) allowing for interactions between the variables was calculated to assess whether *health* and *disease duration* influenced the relationship between ASAS and PAM. Model 3 (adjusted R^2^ = 0.426, F(19, 187) = 9.03, *p* < 0.0001, AIC = 1234.65) revealed no significant interactions between the ASAS and disease duration (*p* = 0.899), or the different levels of health (*p* = 0.987, 0.851, 0.975, 0.986, respectively). Therefore, the association between the ASAS-R and the PAM remains irrespective of sociodemographic or health-related data.

## 4. Discussion

The aim of the current study was to assess the validity and factor structure of the German translation of the ASAS-R. For this purpose, *n* = 215 datasets of community-dwelling people with chronic illness were analyzed regarding psychometric measures. Convergent validity was tested via comparison with the PAM.

Overall, satisfactory correlation between ASAS-R and PAM suggests good convergent validity, indicating that the translated version of the ASAS is able to capture the desired construct well. The correlation of the ASAS-R and the PAM was 0.46, which is comparable to the convergent validity levels found in other translation studies for self-management questionnaires [24,33,49,64]. As self-care and self-management are highly complex, individual constructs that have not yet been universally defined [3], we expected a medium correlation between both questionnaires [52], especially as both questionnaires cover different aspects of this complex behavior [48]. Likewise, the regression analyses identified the ASAS-R as a significant predictor of the PAM irrespective of other sociodemographic and health-related variables. These results indicate good convergent validity, considering that the two questionnaires measure similar but not the same constructs [24,32].

The German ASAS-R revealed no floor but medium ceiling effects for several items, indicating that some items were not sufficient for our participants. Of note, floor and ceiling effects were not reported in the original assessment of the ASAS-R [28]; therefore, no comparison can be made here to assess whether the ceiling effects are specific to our translation. However, many other questionnaires on self-management and related constructs have reported ceiling effects [33,34,65,66]. Of note, it is important to mention that our study sample consisted of community-dwelling adults, most of which were only moderately restricted in their ADLs. In addition, the participants had an average disease duration of 13.8 years, meaning they had had enough time to develop and practice self-management. Therefore, it is plausible that in our group of participants, most people scored high on the ASAS-R items, indicating that the items were indeed able to capture their situation correctly. Still, it is necessary to test the questionnaire in other populations, such as older, physically impaired people, or in people newly diagnosed with illnesses to assess initial self-management.

Although the overall ASAS-R revealed good internal consistency in our analysis, the original ASAS-R publication suggested three underlying factors, namely ‘*having* power for self-care’, ‘*developing* power for self-care’, and ‘*lacking* power for self-care’ [28]. However, CFA revealed a poor fit of this structure for our data. Subsequent PCA again revealed three factors, but the item attribution to those factors differed from the original ASAS-R publication. Of note, the initial ASAS-R as proposed by Evers et al. [29] before the revision by Sousa et al. [28] did not specify a particular factor structure but rather suggested an overall sum score, with higher factors indicating higher levels of self-care agency. In addition, a preliminary study by Sousa et al. [67] that preceded the revised ASAS version also identified a single factor. The authors explained this inconsistency in their own revised version by taking into account the different samples used for analysis [28]. In other studies assessing the factor structure of the ASAS, ASAS-R, or translated versions, one Chinese study found a seven-factor solution [68], while Söderhamn and Cliffordson [31] found five underlying factors in a Swedish sample. To validate their Spanish translation, Stacciarini and Pace [69] only used CFA to confirm the three-factor structure as proposed by Sousa et al. (2011) [28], but did not perform PCA to assess whether a different model fit their data better. However, their overall factor loadings and internal consistency of the factors were comparable to the present results, regardless of the different item attribution. Of note, they only had a sample size of 150 people, while a Spanish translation by Colomer-Pérez and Useche [70] on 900 students revealed four factors. Overall, these results suggest that the factor structure of the ASAS and its revised or translated versions is not universal and differs between datasets. One possible explanation for these differences is the highly complex and individual nature of self-management itself, which may vary depending on a variety of factors such as the disease and its duration, physical and mental health, and sociodemographic variables [1,3,8]. Due to this complexity, certain factors may play a role for self-management for some people that do not matter to others. Likewise, the literature shows different definitions of self-management, including many different sub-factors and facets. Therefore, it is not yet possible to derive a universally accepted structure of self-management [3,4,8]. As a consequence, we suggest the individual assessment of the given factor structure in each dataset to take into account the specific characteristics of each sample of participants. Overall, we therefore conclude that the differing factor structure in our analysis is not a sign of a deficient questionnaire translation, but instead a consequence of the highly complex concept the questionnaire aims to measure.

Overall, the ASAS was derived based on Orem’s Theory of Self-Care [25] and covers an important aspect of healthcare, namely self-care and, as an extension of self-care, self-management. As people continuously grow older and the relevance of chronic illness increases, it becomes crucial to reliably assess people’s self-management abilities in order to relieve healthcare systems and enable high-quality care [1,18,71]. Due to the high complexity and individual nature of self-management, no single questionnaire has been developed that covers all important aspects; therefore, a mixture of questionnaires is recommended for the assessment of self-management [4]. The ASAS proves to be a useful addition to this collection, as it is less hypothetical than other questionnaires and therefore an approximation of the behavior rather than solely the confidence in self-management [33,48].

### Limitations

Our study is not free of limitations. We included community-dwelling adults with at least one chronic illness to assess the applicability of the questionnaire for the general public; however, this reduces the generalizability to an acutely ill patient population or to newly diagnosed patients. Likewise, the long average disease duration in the present participants may have influenced the responses to the ASAS-R and resulted in the reported ceiling effects. The questionnaire should therefore be tested again in different populations, especially in newly diagnosed patients, to assess the initial development of self-management.

Due to the COVID-19 pandemic, data collection was performed online, which may have influenced data quality due to attentional deficits. However, we took precautions to ensure best possible data quality as described in the Methods section. In contrast to attentional effects, the use of a fully anonymous online survey may have reduced social desirability and bias compared to face-to-face testing. In addition, although the information on the study was available to all patients in the hospital without active selection bias from the authors, the online recruitment of patients may have introduced a bias toward highly educated and comparably healthy patients, as it required participants to own a smartphone or computer.

Lastly, as Greene et al. (2022) [72] reported, model fit and fit measures are highly dependent on the type of data and the factor analysis method. Therefore, the deviation of our item attribution from the original ASAS structure by Sousa et al. (2011) and other previous publications must be interpreted in light of the methods and participants included in the respective studies. As Greene et al. (2022) [72] indicated, model-based fit measures should not be seen as ultimate but instead be interpreted with regards to content and theoretical frameworks. In our analysis, we therefore not only looked at the best model fit but also decided that our variable attribution seems more reasonable in terms of actual content of the identified factors. For example, it makes more sense that the item 8 “in the past, I have changed some of my old habits in order to improve my health” is assigned to the factor *having* power for self-care rather than *developing* power for self-care, as indicated in the original publication [35]. Overall, our analysis, as well as previous studies, suggest the need to individually assess the questionnaire structure for each dataset in future analyses, especially as self-management is such a complex construct that it may well vary depending on the participant group, country, and diagnoses [3].

## 5. Conclusions

The German translation of the ASAS-R showed good psychometric properties and convergent validity, indicating that it was able to capture the construct of self-management well. However, as floor and ceiling effects were present, the questionnaire should be tested again in a population of severely ill or newly ill patients to see whether it can capture new-onset self-management. Of note, the factor structure underlying the questionnaire differed from the original publication, and different factor structures have been identified in earlier studies. Therefore, it is recommended to individually assess the factor structure for each dataset. As stated in previous literature and again shown in our analysis, both self-care and subsequently self-management are highly complex and individual constructs; therefore, they should be assessed with multiple measures to capture all relevant aspects. Based on our analysis, the German ASAS-R can now be used to approach these important aspects of healthcare in German-speaking populations.

## Figures and Tables

**Table 1:** Sociodemographic information.

Variable	M (SD)	Median (IQR)	Range	*n*
Age	51.633 (14.686)	47 (59–42)	92–19	215
Number of Medications	3.219 (2.802)	3 (4–1)	18–0	215
Number of Diagnoses	1.986 (1.190)	2 (2–1)	8–1	215
Disease Duration	13.778 (11.071)	12 (20–5)	57–0	207
ASAS-R Sum	52.805 (8.386)	53 (58–48)	72–20	215
PAM Sum	40.576 (5.925)	40 (45–37)	52–20	215
	**Value**	**Count**	**%**	***n***
Gender				215
	Male	64	29.767	
	Female	151	70.233	
Education				214
	Low	16	7.477	
	Medium	110	51.402	
	High	88	41.121	
Marital State				215
	Married	139	64.651	
	Single	50	23.256	
	Divorced/Widowed	26	12.093	
Current Health				215
	1 Excellent	5	2.326	
	2 Very Good	29	13.488	
	3 Good	78	36.279	
	4 Not Good	90	41.860	
	5 Poor	13	6.047	
ADL				215
	1 Very restricted	46	21.395	
	2 A Bit Restricted	113	52.558	
	3 Not Restricted	56	26.047	

Note: ADL = activities of daily living, ASAS = Appraisal for Self-Care Agency Scale-Revised, PAM = Patient Activation Measure.

**Table 2:** Properties of the ASAS-R items (*n* = 215).

				Response Frequencies						
Item	r *	cor.r *	Mean (SD)	1	2	3	4	5	Floor	Ceiling
1	0.59	0.67	4.2 (0.85)	0.01	0.05	0.08	0.47	0.39	2 (0.9%)	83 (17.7%)
2	0.63	0.74	4.0 (0.83)	0.01	0.04	0.13	0.54	0.27	3 (1.4%)	58 (27.0%)
3	0.60	0.69	4.1 (0.76)	0.00	0.04	0.10	0.56	0.29	1 (0.5%)	63 (29.3%)
4	0.40	0.43	2.6 (1.31)	0.22	0.35	0.10	0.24	0.09	48 (22.3%)	19 (8.8%)
5	0.31	0.38	3.8 (0.98)	0.02	0.10	0.18	0.46	0.25	4 (1.9%)	53 (24.7%)
6	0.56	0.59	3.5 (1.13)	0.03	0.24	0.12	0.43	0.19	6 (2.8%)	40 (18.6%)
7	0.39	0.44	3.4 (1.27)	0.07	0.23	0.12	0.34	0.23	16 (7.4%)	50 (23.5%)
8	0.43	0.50	3.7 (1.00)	0.03	0.11	0.21	0.45	0.20	6 (2.8%)	44 (20.5%)
9	0.31	0.41	3.7 (0.91)	0.02	0.08	0.27	0.47	0.15	5 (2.3%)	33 (15.3%)
10	0.48	0.58	3.6 (0.88)	0.01	0.12	0.29	0.46	0.12	2 (0.9%)	26 (12.1%)
11	0.48	0.46	2.7 (1.11)	0.11	0.41	0.18	0.25	0.05	23 (10.7%)	11 (5.1%)
12	0.33	0.39	4.1 (0.89)	0.01	0.06	0.08	0.48	0.37	3 (1.4%)	79 (36.7%)
13	0.38	0.40	3.9 (0.98)	0.02	0.11	0.09	0.54	0.24	5 (2.3%)	51 (23.7%)
14	0.46	0.50	3.0 (1.26)	0.13	0.30	0.18	0.27	0.13	27 (12.6%)	27 (21.6%)
15	0.47	0.51	2.5 (1.26)	0.22	0.38	0.16	0.15	0.10	47 (21.9%)	21 (9.8%)
	**A**	**Std. α**	**Mean (SD)**	**95% CI**			**Range**		**Floor**	**Ceiling**
Sum	0.82	0.83	52.81 (8.39)	0.79–0.86			72–20		0 (0%)	0 (0%)
Fac1	0.80	0.80	2.90 (0.91)	0.76–0.85			25–5		2 (0.93)	2 (0.93)
Fac2	0.78	0.79	4.0 (0.63)	0.73–0.82			25–5		1 (0.47)	11 (5.12)
Fac3	0.67	0.69	3.60 (0.72)	0.59–0.74			20–4		1 (0.47)	8 (3.72)

* Note: r = item-total-correlation with item itself excluded, cor.r = item-total correlation corrected for item overlap and scale reliability [51], Fac1-Fac3 = PCA-based factors 1 to 3.

**Table 3:** Confirmatory factor analysis for the current model.

Model Fit						
Model	𝜒^2^	Df		*p*		
Baseline	1119.79	91				
Factor Model	155.563	74		<0.001		
**CFI**	**TLI**	**AIC**	**RMSEA**	**95% CI**	***p***	
0.921	0.903	7721.316	0.072	0.056, 0.087	0.014	
**Parameter Estimates**						
**Factor**	**Item**	**Est. (ß)**	**Std. Error**	**z-value**	*p*	**95% CI**
Factor 1	ASAS1	0.614	0.053	11.607	<0.001	0.510, 0.717
	ASAS2	0.721	0.048	14.980	<0.001	0.627, 0.815
	ASAS3	0.602	0.046	13.217	<0.001	0.513, 0.692
	ASAS8	0.511	0.068	7.550	<0.001	0.378, 0.644
	ASAS12	0.370	0.061	6.023	<0.001	0.250, 0.490
Factor 2	ASAS5	0.496	0.069	7.164	<0.001	0.360, 0.632
	ASAS7	0.516	0.092	5.631	<0.001	0.336, 0.696
	ASAS9	0.628	0.061	10.236	<0.001	0.508, 0.748
	ASAS10	0.748	0.057	13.016	<0.001	0.635, 0.861
Factor 3	ASAS4	0.684	0.088	7.738	<0.001	0.510, 0.857
	ASAS6	0.689	0.074	9.318	<0.001	0.544, 0.384
	ASAS11	0.633	0.074	8.579	<0.001	0.488, 0.777
	ASAS14	1.075	0.074	14.467	<0.001	0.929, 1.221
	ASAS15	1.020	0.076	13.459	<0.001	0.871, 1.168

**Table 4:** Linear regression on PAM total score.

	PAM		
Predictors	Est. (ß)	CI	*p*
(Intercept)	31.76	24.49–39.04	**<0.001**
ASAS-R	0.28	0.20–0.36	**<0.001**
Age	−0.00	−0.06–0.05	0.875
Number of Diagnoses	−0.55	−1.21–0.10	0.099
Disease Duration	0.07	0.01–0.13	**0.020**
Gender: Male	0.54	−0.96–2.04	0.479
Education: Medium	1.98	−0.59–4.54	0.130
Education: High	3.66	1.05–6.27	**0.006**
Health: Very good	−7.20	−11.61–−2.80	**0.001**
Health: Good	−7.39	−11.66–−3.13	**0.001**
Health: Not good	−10.09	−14.43–−5.75	**<0.001**
Health: Poor	−13.65	−19.00–−8.30	**<0.001**
ADL: Lightly Restricted	−0.01	−1.90–1.88	0.991
ADL: Not Restricted	0.36	−1.99–2.71	0.761
Number of Medications	0.13	−0.16–0.42	0.381

*n* = 206, R^2^/R^2^ adjusted = 0.476/0.438, AIC = 1220.65, ADL = Activities of Daily Living, ASAS-R = Appraisal for Self-Care Agency Score Revised, CI = 95% Confidence Intervals, PAM = Patient Activation Measure. Significant values are accentuated in bold.

## Data Availability

The anonymous data are freely available from: Schönenberg, A., Prell, T., Teschner, U., & Mühlhammer, H. M. (2022). The German version of the Appraisal of Self-Care Agency Scale-Revised (ASAS-R), OSF. 10.17605/OSF.IO/WM89J.

## Supplementary Materials

The following supporting information can be downloaded at: https://www.mdpi.com/article/10.3390/healthcare10091785/s1, Table S1: Translated questionnaire, Table S2: Overview of Diagnoses, Table S3: Confirmatory Factor Analysis of original factor structure, Table S4: Principal Component Analysis, Table S5: Item-total correlation for all three factors, Figure S1: Correlation Matrix for all ASAS items, Figure S2: CFA Model Plot, Figure S3: Comparison of item attribution to the three factors in original and translated version, Figure S4: Correlation of ASAS and PAM.
