# Supplementary material for: Validation and Psychometric Analysis of the German Translation of the Appraisal of Self-Care Agency Scale-Revised
Source: Healthcare (Basel). 2022 Sep 16;10(9):1785. doi: 10.3390/healthcare10091785 (PMC9498910; doi:10.3390/healthcare10091785)

Supplementary Materials for: The German version of the Appraisal of Self-Care Agency Scale-Revised (ASAS-R)

Supplement Table S1. Translated questionnaire 2

Supplement Table S2: Overview of Diagnoses 3

Supplement Table S3. Confirmatory Factor Analysis of original factor structure 3

Supplement Table S4. Principal Component Analysis 4

Supplement Table S5. Item-total correlation for all three factors 5

Supplement Figure S1. Correlation Matrix for all ASAS items 6

Supplement Figure S2. CFA Model Plot 7

Supplement Figure S3. Comparison of item attribution to the three factors in original and translated version 7

Supplement Figure S4. Correlation of ASAS and PAM items 8

Supplement Table S1. Translated questionnaire.

Im folgenden Abschnitt möchten wir von Ihnen gerne erfahren, wie Sie mit Ihrer Erkrankung umgehen und wie sicher Sie sich dabei fühlen.

Bitte kreuzen Sie für jede der folgenden Aussagen ein Kästchen an, das für Sie persönlich am besten beschreibt, wie sehr Sie der Aussage zustimmen oder nicht zustimmen. Es gibt keine richtigen oder falschen Antworten. Ihre Antworten sollten Ihre persönliche Meinung widerspiegeln, unabhängig davon, was Ihr Arzt gerne von Ihnen hören würde.

	stimme überhaupt nicht zu	stimme nicht zu	Weder noch	stimme zu	stimme voll und ganz zu
1. Wenn sich die Umstände verändern, führe ich nötige Maßnahmen durch, um gesund zu bleiben	❑	❑	❑	❑	❑
2. Wenn sich meine Beweglichkeit verschlechtert, führe ich entsprechende Anpassungen durch	❑	❑	❑	❑	❑
3. Wenn nötig, setze ich neue Prioritäten bei den Maßnahmen, die ich für meine Gesundheit ergreife	❑	❑	❑	❑	❑
4. Mir fehlt oft die Energie, um mich so um mich selbst zu kümmern, wie ich sollte	❑	❑	❑	❑	❑
5. Ich suche nach neuen Möglichkeiten, mich besser um mich selbst zu kümmern	❑	❑	❑	❑	❑
6. Wenn ich es brauche, schaffe ich es, mir Zeit für mich selbst zu nehmen	❑	❑	❑	❑	❑
7. Wenn ich neue Medikamente einnehme, suche ich Informationen über mögliche Nebenwirkungen heraus, um mich besser um mich selbst kümmern zu können	❑	❑	❑	❑	❑
8. In der Vergangenheit habe ich meine alten Gewohnheiten verändert, um meine Gesundheit zu verbessern	❑	❑	❑	❑	❑
9. Ich führe regelmäßig Maßnahmen durch, um die Sicherheit von mir selbst und meiner Familie zu gewährleisten	❑	❑	❑	❑	❑
10. Ich überprüfe regelmäßig die Wirksamkeit von den Maßnahmen, die ich für eine bessere Gesundheit durchführe	❑	❑	❑	❑	❑
11. Bei meinen täglichen Aktivitäten nehme ich mir selten Zeit, mich um mich selbst zu kümmern	❑	❑	❑	❑	❑
12. Ich bin in der Lage, mir selbst Informationen zu beschaffen, wenn meine Gesundheit bedroht ist	❑	❑	❑	❑	❑
13. Wenn ich mich nicht um mich selbst kümmern kann, suche ich mir Hilfe	❑	❑	❑	❑	❑
14. Ich habe selten Zeit für mich selbst	❑	❑	❑	❑	❑
15. Ich kann mich nicht immer so um mich kümmern, wie ich es gerne tun würde	❑	❑	❑	❑	❑

Sousa, V. D., Zauszniewski, J. A., Bergquist-Beringer, S., Musil, C. M., Neese, J. B., & Jaber, A. F. (2010). Reliability, validity and factor structure of the Appraisal of Self-Care Agency Scale-Revised (ASAS-R). *Journal of evaluation in clinical practice*, *16*(6), 1031–1040. https://doi.org/10.1111/j.1365-2753.2009.01242.x

Supplement Table S2: Overview of Diagnoses.

**Diagnosis**	**Count**
Hypertension	75
Other/Miscellaneous	65
COPD	38
Diabetes	34
Mental Illness	29
Arthritis	23
Rheumatic Illness	22
High Cholesterol	21
Heart Attack	17
Thyroid Problems	17
Cancer	14
Osteoporosis	13
Parkinson's Disease	12
Migraines	11
Chronic Kidney Disease	8
Multiple Sclerosis	7
Stroke/other form of circulatory disorder	7
Fractures	6
Long COVID	5
Cataracts	2

Supplement Table S3. Confirmatory Factor Analysis of original factor structure.

**Model**	X^2	**Df**		***p***	
Baseline	1170.64	105			
Factor Model	302.689	87		<.001	
**CFI**	**TLI**	**AIC**	**RMSEA**	**95 % CI**	***p***
.798	.756	8420.25	.107	.094, .121	< .001

Supplement Table S4. Principal Component Analysis.

Chi² Test				
	**Value**	**Df**	**p**	
	191.652	63	< .001	
**Component Loadings**				
**Variable**	**Component 1**	**Component 2**	**Component 3**	**Uniqueness**
ASAS14			0.886	0.226
ASAS15			0.779	0.314
ASAS11			0.727	0.459
ASAS6			0.654	0.464
ASAS4			0.488	0.516
ASAS3	0.849			0.281
ASAS2	0.776			0.282
ASAS1	0.723			0.391
ASAS12	0.620			0.644
ASAS8	0.461			0.597
ASAS9		0.783		0.387
ASAS10		0.750		0.317
ASAS7		0.565		0.622
ASAS5		0.463		0.575
ASAS13				0.767
Component Characteristics				
	**Unrotated Solution**		**Rotated Solution**	
**Component**	**Eigenvalue**	**Cumulative Variance**	**Sumsq. Loadings**	**Cumulative Variance**
1	4.664	0.311	2.903	0.194
2	2.399	0.471	3.010	0.394
3	1.096	0.544	2.247	0.544

Supplement Table S5. Item-total correlation for all three factors.

Factor	Item	r*	cor. r*	Mean (SD)	Inter-item-corr
1	1	.59	.69	4.2 (.85)	.38
	2	.72	.83	4.0 (.83)	
	3	.70	.80	4.1 (.76)	
	8	.44	.50	3.7 (1.0)	
	12	.37	.42	4.1 (.89)	
2	5	.41	.49	3.8 (.98)	.35
	7	.35	.42	3.4 (1.3)	
	9	.49	.66	3.7 (.91)	
	10	.61	.77	3.6 (.88)	
3	4	.48	.54	2.6 (1.3)	.44
	6	.55	.61	3.5 (1.1)	
	11	.50	.56	2.7 (1.1)	
	14	.73	.83	3.0 (1.3)	
	15	.70	.79	2.5 (1.3)	

Supplement Figure S1. Correlation Matrix for all ASAS items.

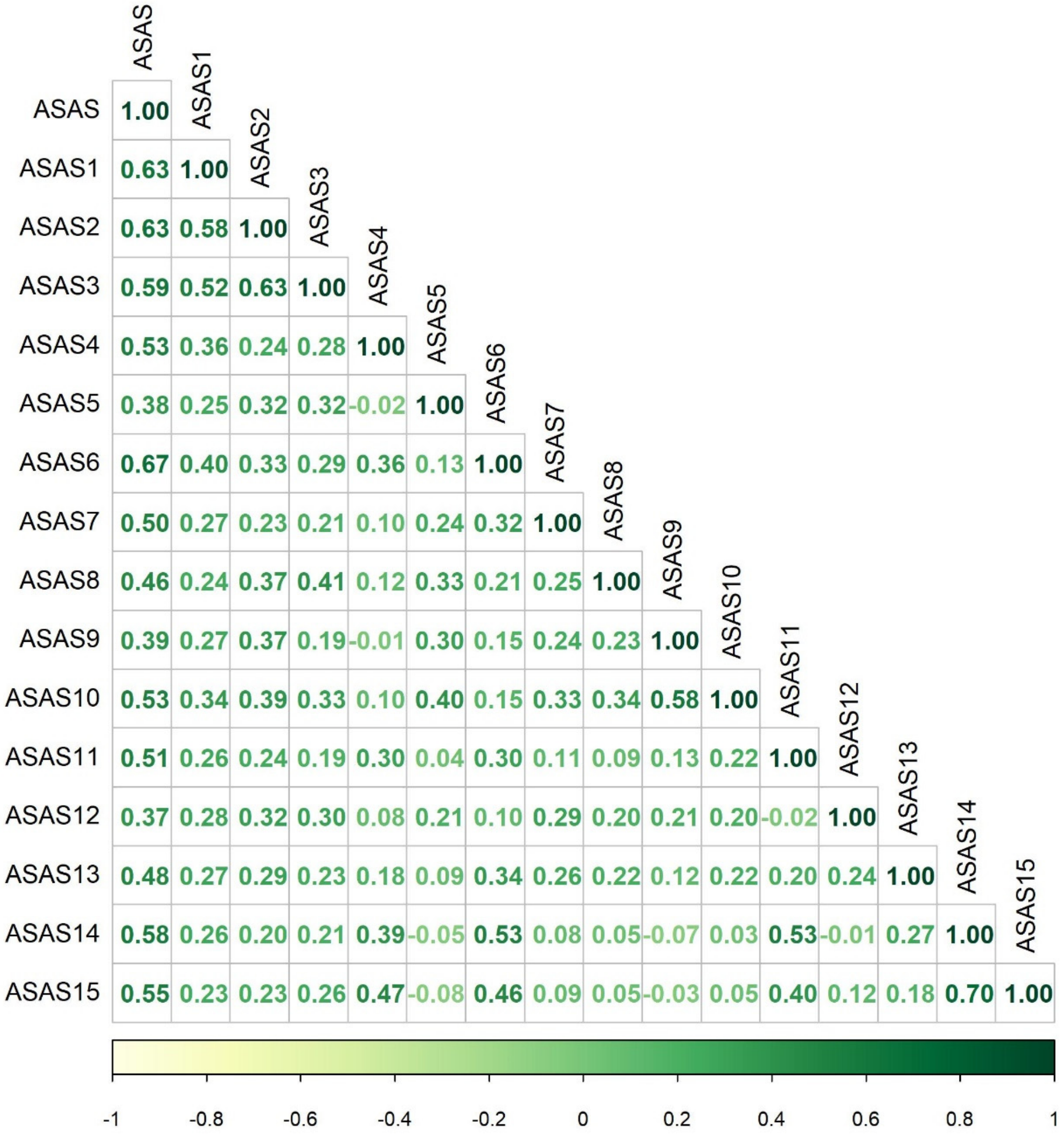

Supplement Figure S2. CFA Model Plot.

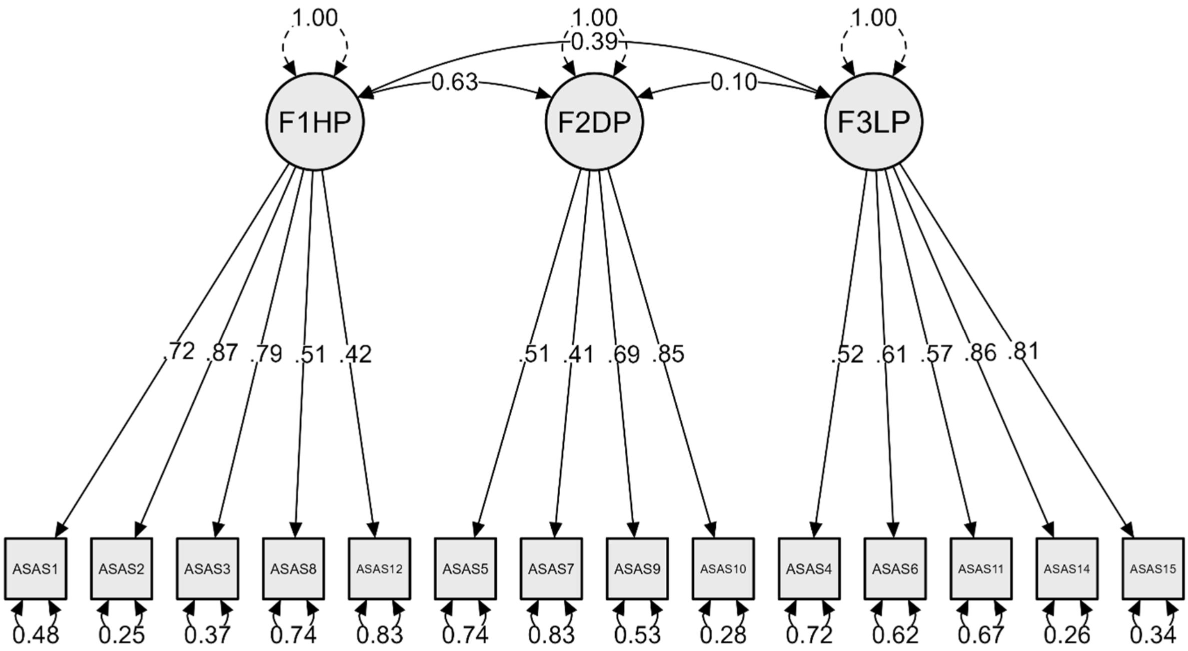

Note: F1HP = Factor 1 Having Power for Self-Care, F2DP = Factor 2, Developing Power for Self-Care, F3LP = Factor 3 Lacking Power for Self-Care

Supplement Figure S3. Comparison of item attribution to the three factors in original and translated version.

Original

Factor 1: *Having* Power for Self-Care

1. As circumstances change, I make the needed adjustments to stay healthy
2. If my mobility is decreased, I make the needed adjustments
3. When needed, I set new priorities in the measures that I take to stay healthy
5. I look for better ways to take care of myself
6. When needed, I manage to take time to care for myself
10. I regularly evaluate the effectiveness of things that I do to stay healthy

Factor 2: *Developing* Power for Self-Care

7. If I take a new medication, I obtain information about the side effects to better care for myself
8. In the past, I have changed some of my old habits in order to improve my health
9. I routinely take measures to insure the safety of myself and my family
12. I am able to get information I need, when health is threatened
13. I seek help when unable to care for myself

Factor 3: *Lacking* Power for Self-Care

4. I often lack energy to care for myself in the way that I know I should
11. In my daily activities I seldom take time to care for myself
14. I seldom have time for myself
15. I am not always able to care for myself in a way I would like

Translation

Factor 1: *Having* Power for Self-Care

1. As circumstances change, I make the needed adjustments to stay healthy
2. If my mobility is decreased, I make the needed adjustments
3. When needed, I set new priorities in the measures that I take to stay healthy
8. In the past, I have changed some of my old habits in order to improve my health
12. I am able to get information I need, when health is threatened

Factor 2: *Developing* Power for Self-Care

5. I look for better ways to take care of myself
7. If I take a new medication, I obtain information about the side effects to better care for myself
9. I routinely take measures to insure the safety of myself and my family
10. I regularly evaluate the effectiveness of things that I do to stay healthy

Factor 3: *Lacking* Power for Self-Care

4. I often lack energy to care for myself in the way that I know I should
6. When needed, I manage to take time to care for myself
11. In my daily activities I seldom take time to care for myself
14. I seldom have time for myself
15. I am not always able to care for myself in a way I would like

Unclassified: 13. I seek help when unable to care for myself

Supplement Figure S4. Correlation of ASAS and PAM items.

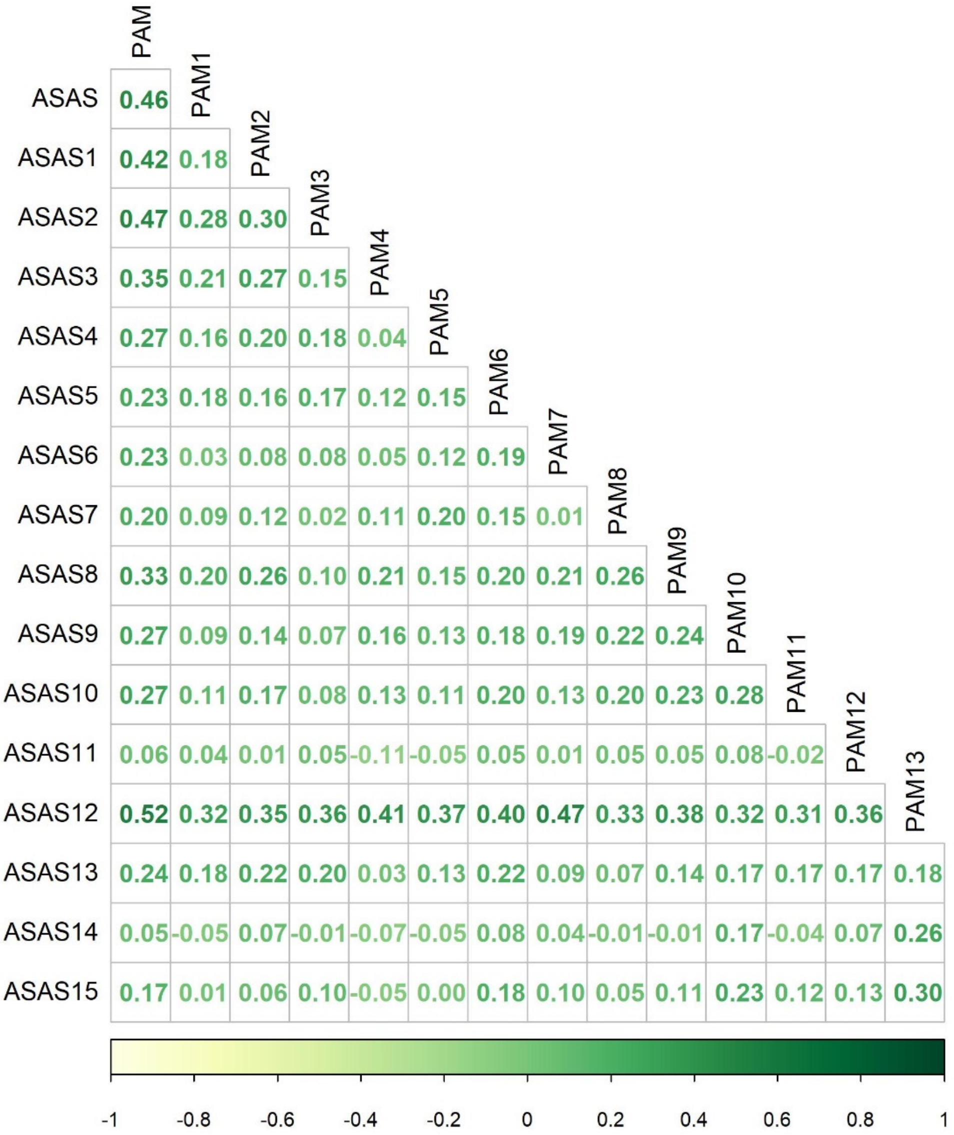

Supplement: Supplementary file 1 [file healthcare-10-01785-s001.zip › healthcare-1885004-supplementary.pdf]
